# A Smartphone Game-Based Intervention (Tumaini) to Prevent HIV Among Young Africans: Pilot Randomized Controlled Trial

**DOI:** 10.2196/10482

**Published:** 2018-08-01

**Authors:** Kate Winskell, Gaëlle Sabben, Victor Akelo, Ken Ondeng'e, Christopher Obong'o, Rob Stephenson, David Warhol, Victor Mudhune

**Affiliations:** ^1^ Rollins School of Public Health Hubert Department of Global Health Emory University Atlanta, GA United States; ^2^ Kenya Medical Research Institute- Centre for Global Health Research HIV Research Branch Kisumu Kenya; ^3^ School of Public Health University of Memphis Memphis, TN United States; ^4^ School of Nursing and The Center for Sexuality and Health Disparities University of Michigan Ann Arbor, MI United States; ^5^ Realtime Associates El Segundo, CA United States

**Keywords:** HIV, youth, Sub-Saharan Africa, Kenya, serious game, narrative, smartphone, pilot study, randomized controlled trial, mhealth, prevention

## Abstract

**Background:**

There is a pressing need to ensure that youth in high HIV prevalence settings are prepared for a safer sexual debut. Smartphone ownership is increasing dramatically in low-income and middle-income countries. Smartphone games that are appropriately grounded in behavioral theory and evidence-based practice have the potential to become valuable tools in youth HIV prevention efforts in Sub-Saharan Africa.

**Objective:**

To pilot-test a theory-based, empirically grounded smartphone game for young Kenyans designed to increase age and condom use at first sex, aiming to establish directionality of effects on behavior change.

**Methods:**

Tumaini (“hope for the future” in Swahili) is an interactive, narrative-based game grounded in social cognitive theory. A randomized controlled pilot study was conducted in Kisumu, Western Kenya, from April to June 2017 with 60 participants aged 11-14 (mean 12.7) years. Intervention arm participants (n=30) were provided with an Android smartphone with Tumaini installed on it and were instructed to play the game for at least 1 hour a day for 16 days; control arm participants (n=30) received no intervention. All participants completed a survey on behavioral mediators, delivered via an audio computer-assisted self-interview system at baseline (T1), post intervention (T2), and at 6 weeks postintervention (T3). The postintervention survey for intervention arm participants included questions eliciting feedback on the game. Intervention arm participants and their parents participated in 8 postintervention focus group discussions. Game log files were analyzed to calculate the length of exposure to the game. Behavioral survey data were analyzed using two-sample *t* tests to compare mean change from T1 to T2 and to T3 for intervention versus control arm participants. Descriptive statistics on game feedback questions were computed. Focus group transcripts were uploaded to MAXQDA software, where they were labeled with deductive and inductive codes. Data were analyzed thematically and compared across demographics.

**Results:**

Intervention arm participants played Tumaini for a mean of approximately 27 hours. The intervention arm showed significant gains in sexual health-related knowledge and self-efficacy (both *P*<.001), behavioral intention for risk-avoidance strategies and sexual risk communication (*P*=.006), and overall survey scores (*P*<.001) compared with the control arm at T3. The postintervention survey revealed high subjective measures of the game’s value, relevance, and appeal. Focus groups identified a wide range of knowledge and skills the participants had gained, including setting goals and planning how to achieve them, which was perceived as a key motivator for avoiding or reducing risk.

**Conclusions:**

The study supports the need for further research to assess the efficacy of the game-based intervention. If proven efficacious, smartphone games have the potential to dramatically increase the reach of culturally adapted behavioral interventions while ensuring fidelity to intervention design.

**Trial Registration:**

ClinicalTrials.gov NCT03054051; http://clinicaltrials.gov/ct2/show/NCT03054051 (Archived by WebCite at http://www.webcitation.org/70U2gCNtW)

## Introduction

A third of all new adult HIV infections occur in young people aged 15-24 years [1]. In African countries most affected by HIV, demographic change is increasing the size of adolescent cohorts, thereby increasing their contribution to HIV incidence [2]. In addition, this age group suffers disproportionately high levels of HIV-related morbidity and mortality [3]. Reaching preadolescents with prerisk prevention interventions may help establish lifelong patterns of safer sexual behavior and avert high-risk behaviors in the future [4,5]; for example, those who use condoms at first sex are more likely to use them consistently in the future [6,7].

Electronic games have the potential to be a valuable tool in youth HIV prevention in Sub-Saharan Africa if they are appropriately grounded in behavioral and instructional theory [8,9], informed by existing evidence-based interventions [10], and contextually appropriate. Smartphone ownership is increasing dramatically in emerging and developing nations [11], opening up new possibilities for delivering highly interactive, culturally relevant mHealth interventions at scale and low cost. Serious digital games [12] have high entertainment and motivational appeal for young people. They also have distinctive advantages from the perspective of pedagogy and behavioral theory. By allowing players to experience real agency in a virtual and safe environment, well-designed games provide a level of experiential learning unparalleled by many other interventions. They are particularly well aligned with key constructs of social cognitive theory [13], allowing for both cognitive and behavioral rehearsal through role-play and simulation. Although a relatively limited number of games to date have been designed with solid theoretical grounding and rigorously evaluated [14-20], there is evidence of their effectiveness for health, including clinical, outcomes [21-28].

In addition to their appeal, mobile games for sexual health have further distinctive advantages over common group-based, evidence-based interventions [29]. They have considerable potential for scalability, low cost per person reached, and cultural adaptability. Exposure to the intervention can be reliably measured through automated data collection, which can also help pinpoint “active ingredients,” contributing to the building of behavioral, pedagogical, and game design theory. Fidelity to intervention design is much more likely as the intervention is no longer dependent on a skilled cadre of facilitators. Electronic delivery offers potential for remote updates, while portability via mobile handsets can allow the intervention to link into people’s everyday lives, offering more sustained intervention exposure.

There is a pressing need to assess the feasibility of using game technologies for HIV prevention in low-resource settings and their potential for efficacy. In this study, we pilot-tested an interactive narrative-based smartphone game to prevent HIV among preadolescents in Kisumu Town, Western Kenya, where adult HIV prevalence (19.9%) is over three times the national average [30,31]. We describe here results from this pilot study of the game’s potential to influence behavioral mediators of increased age and condom use at sexual debut.

## Methods

### Study Design

**Figure 1:**
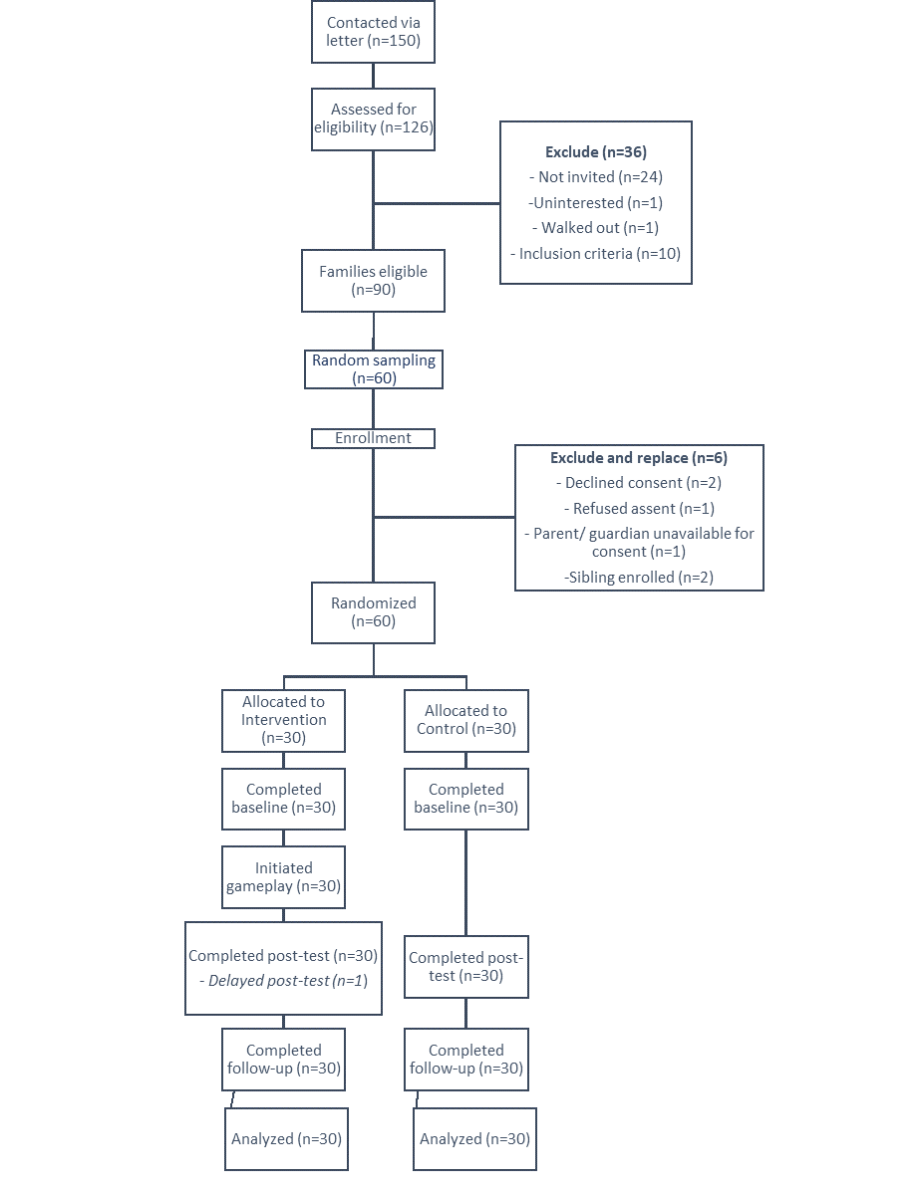
CONSORT flow diagram.

We conducted an individually randomized pilot study of the game *Tumaini* (“hope for the future” in Swahili) in a sample of 60 male and female preadolescents aged 11-14 years in periurban and urban Kisumu, Kenya, between April and June 2017. The intervention was carried out over 16 days during the 3-week school holiday in April 2017 (Figure 1). Assessment was performed via a survey at baseline (T1), immediately postintervention (T2), and at 6 weeks postintervention (T3). Intervention arm participants also took part in focus group discussions (FGDs) after the intervention to provide additional data on the game experience. The study was approved by the Emory University and Kenya Medical Research Institute (KEMRI) Institutional Review Boards and was registered with ClinicalTrials.gov (NCT03054051).

The eligibility criteria for participation were as follows: age 11-14 years, grade 3-4 English proficiency on the Flesch-Kincaid Reading Scale, residence in Kisumu Town, and willingness to complete all study activities. Letters were distributed through schools to parents of age-eligible children inviting them to attend informational meetings. Consent and assent were secured at the home of the participants, following an explanation of the study. Parents consented to participate in the postintervention focus groups if their child was randomized to the intervention arm. No incentives were provided.

### Participants

### Randomization

Participants (n=60) were randomized 1:1 to the control arm (n=30) or the intervention (game) arm (n=30) of the study. Randomization, stratified by the school attended by the participant, gender, and age, was undertaken using a coin flip by a blinded research team member. Within each school, gender, and age block of participants, coin flips were repeated until participants were equally distributed between the two study arms. Assignments were revealed to participants after they had completed the baseline assessment.

### Intervention

Participants assigned to the intervention arm played *Tumaini* (Multimedia Appendix 1), a theoretically grounded, narrative-based game for inexpensive Android smartphones developed in collaboration with a US commercial game developer, Realtime Associates, and with input from US-based and Kenyan specialists in adolescent sexual health and Kenyan preadolescents and their parents.

*Tumaini* is designed to increase age and condom use at first sex by increasing knowledge about sexual health and HIV; building risk-avoidance and risk-reduction skills and related self-efficacy; challenging HIV stigma and harmful gender norms and attitudes; fostering future orientation, goal setting, and planning; and promoting dialogue with adult mentors.

The game’s design draws on social behavioral theory, including social cognitive theory [13] and the theory of possible selves [32]; existing evidence-based interventions for youth HIV prevention [33,34-36]; and games for health [8,12,17,37] and entertainment-education [38] literature. It is grounded in research on HIV-themed narratives written by young Africans [39-41]. *Tumaini* uses interactive narrative to promote observational learning, cognitive and behavioral rehearsal, problem-solving, and immersion.

The game is made up of 3 intersecting components. First, the role-playing narrative (Multimedia Appendix 2) uses a “choose-your-own-adventure” format that allows the player to make decisions for 6 diverse characters and observe the consequences of those choices in the characters’ lives. At the start of the game, the characters are 3 boys and 3 girls aged 11-14 years. Players role-play each of the 6 young characters as they pass into or through adolescence and face real-life challenges that the players are likely to face at some stage in their own lives. These include peer pressure, puberty, violence, and decisions about smoking, alcohol, drugs, and sex. The story takes place over 18 chapters, distributed over 3 levels (Multimedia Appendix 3). For example, in the second level, the player chooses whether to have a male character, Juma, drink alcohol at a party. Later in the chapter, this decision affects Juma, his sexual decision-making, his ability to recognize the risks associated with failing to use a condom, and his options regarding, and successful negotiation of, condom use. These choices regarding sex and condom use have consequences on his sexual health in a subsequent chapter. Second, the mini-games are designed to reinforce knowledge and skills relating to puberty; HIV and other sexually transmitted infections (STIs); pregnancy and avoiding pregnancy; identifying, avoiding, and responding to risk situations; and resisting peer pressure. Mini-games take various forms, including quizzes, jigsaws, and role-playing scenarios with feedback. The topics of the mini-games coordinate with the topics in the role-playing narrative. The third component, *My Story*, incorporates a customizable avatar and invites players to connect the knowledge and skills they learn in the game with their own lives, including through setting goals and how they will achieve them. As with the mini-games, the topics for this part of the game coordinate with the main role-playing narrative.

*Tumaini* comprises approximately 12 hours of discrete gameplay and is designed to be replayed so that players can observe the outcomes of different decisions. Each chapter is accompanied by either a mini-game or a *My Story* component. The player is rewarded with prizes (furniture and other items for the player’s virtual home) upon successful completion of game components. There are 40 possible endings in the role-playing game across the 6 characters. Once the player finishes the last chapter and observes the long-term outcomes for the characters, he or she can replay and collect the remaining prizes by making different choices and observing different outcomes. This rewards system thus encourages players to explore the game and experience the consequences of both health-protective and harmful choices.

Intervention arm participants completed a 45-minute informational onboarding session, including instructions on the interface, technology, and game content. They were instructed to play at least 1 hour per day for the 16 days of the study and asked not to share their own gameplay profile with others. The game interface allows for 5 additional players’ profiles so that others may play without compromising the enrolled player’s data. Intervention participants were provided with a phone with the game preloaded and used it at their own pace for the duration of the intervention. Control participants received standard of care, namely no additional intervention beyond any existing sex education from family, school, and peers. No specific data on the content or source of this education were collected from participants. All study smartphones were returned by the participants at the end of the intervention period.

### Survey Measures

All participants completed a self-administered behavioral survey at T1, T2, and T3. The English language survey was completed at the KEMRI offices, using the audio computer-assisted self-interview (ACASI) system with headphones to protect privacy. It took approximately 1 hour to complete. Surveys were the same for participants in the control and intervention arms for T1 and T3: participants in the intervention arm completed additional survey items at T2, providing subjective assessments of the game itself, including its appeal, value, and relevance.

The behavioral survey assessed mediators associated with age at onset of sexual activity and condom use at sexual debut, including knowledge, self-efficacy, risk assessment, perceived social norms, attitudes, and behavioral intentions. Thematic areas included puberty, sex, relationships, peer pressure, condom use, HIV, STIs, pregnancy, and alcohol and drugs. Table 1 provides examples of the questions and response options by theoretical construct.

**Table 1:** Behavioral survey measures: sample questions and response options by theoretical construct.

Theoretical construct	Sample questions	Response options	Thematic area
Knowledge	*Can a girl get pregnant the first time she has sex?*	Yes No	Pregnancy
Attitudes	*There are times when it is ok to force someone to have sex.* Do you think this is true?	Yes Maybe No	Sex
Self-efficacy	*If you have a question about puberty, how sure are you that you could ask someone you trust for advice?*	Very sure A little sure Not sure	Puberty
Intention	*Imagine that in several years you are in a couple.* Would you talk with your partner about preventing HIV?	Yes No	Condoms
Perceived social norms	“*Most young people like me would not want to be friends with someone who has HIV.”* Do you think this is true?	Yes Maybe No	HIV
Sources of advice	*If you have a question about sex, who will you talk to?*	Mother Father Brother/sister Grandmother/grandfather Aunt /uncle Friend Religious leader Teacher Doctor or nurse A peer educator Someone else No one	Sex

Where possible, items were drawn from existing surveys with a focus on instruments that had been validated in Sub-Saharan African youth populations [33,42-52]. Source literature for these measures included the tool used as part of the evaluation of the Families Matter! Program (FMP) with a similar Nyanza youth population [52]; measures validated with adolescents in Botswana as part of a randomized controlled trial (RCT) of Project AIM [33] conducted by CDC’s Division of Global HIV and TB; the questionnaire from the Guttmacher Institute’s Protecting the Next Generation Project, used with adolescents in Uganda [42]; Kalichman et al’s brief HIV stigma scale, validated in South Africa [43]; IMB subscales (including, eg, the Perceived Effectiveness of AIDS Preventive Behavior sub-scale) [44], used with adolescents in South Africa; the Gender Equitable Men (GEM) Scale [47]; and CARE’s Gender Equity Index (GEI; modeled after the GEM scale for 10-14 year olds and tested and implemented in Kenya) [45]. Items were adapted, where necessary, to be culturally, linguistically, and age-appropriate and consistent in formatting. A draft instrument was presented to parents for acceptability, then cognitively tested in 3 rounds with preadolescents to ensure acceptability, consistent interpretation, and face validity of the questions. This resulted in, for example, our abandoning 5-point scales as too complex for very young adolescents with limited English proficiency. Also in the interests of age-appropriateness, we included three hypothetical risk scenarios presented as vignettes [53] that contextualized situational risk assessment, behavioral intention, and self-efficacy (see Table 2). The final draft of the instrument was pilot-tested with preadolescents.

**Table 2:** Behavioral survey measures: example of scenario-based question. The Vignette: *Imagine that a boy/girl you like invites you to his/her house after school. He/She tells you that the two of you will be alone.*

Theoretical construct	Questions	Response options
Risk assessment	*How safe is this situation?*	Safe A little unsafe Very unsafe
Intention to avoid risk	*Would you go with him/her?*	Yes No
Self-efficacy for risk avoidance	*If you did not want to go, how sure are you that you could say no firmly?*	Very sure A little sure Not sure

*Knowledge* questions (15 items) focused on puberty, pregnancy, HIV and STIs, alcohol and drugs, and condoms, including procedural knowledge for condom use. These items drew on measures from Save the Children’s Adolescent Puberty Workbook assessment questionnaire [51], the Guttmacher Institute’s Protecting the Next Generation Project survey [42], Kalichman et al’s brief HIV stigma scale [43], and Catania et al’s instrument from an assessment of the prevalence of AIDS-related risk factors [48]. The *self-efficacy* domain (9 items) drew from the measures used in the evaluation of the Families Matter! Program (FMP) [52], Save the Children’s questionnaire [51], Johnson-Mallard’s assessment of women’s self-efficacy [50], and Fisher et al’s IMB subscales [44]. This domain included questions about seeking advice about puberty, refusing to engage in risky situations, using a condom, and discussing HIV and pregnancy prevention with a partner; some questions linked to the hypothetical scenarios were presented as short vignettes, which also included measures of behavioral intention and risk assessment. In addition to the scenario-based questions, *behavioral intention* items (6 items) included measures of intention to communicate with a partner about preventing HIV or pregnancy. *Risk assessment* (4 items) also included an item about unprotected sex. Participants were asked to identify whom they would talk to about puberty, relationships, or sex from a list of individuals they might identify as trusted *sources of advice.* This drew on similar questions used for the evaluation of FMP [52]. *Attitude* questions (15 items) were related to control over one’s future, sex, condom use, HIV, and gender. These items were drawn from Kalichman et al’s scales [43], Catania et al’s measures [48], the GEM scale [47], CARE’s GEI [45], the evaluation of FMP [52], Norris et al’s Sexual Abstinence Behavior Scale [49], and the questionnaire used in the evaluation of Project AIM by CDC’s Division of Global HIV and TB [33]. The thematic areas addressed by the *perceived social norms* (6 items) questions were sex, condom use, HIV, and gender. These measures were adapted from items in Norris et al’s [49] and Kalichman et al’s [43] scales, and the GEM [47] and GEI [45] scales.

Where necessary, definitions of certain terms (including “condom” and “sex”) were included for clarity. The survey also collected demographic information at baseline: age, religion, living situation, school grade, access to computers and smartphones, and proxies for economic status (materials from which home was constructed and number of rooms in the house). ACASI survey data were downloaded as comma-separated values files and compiled into an Excel file for cleaning and analysis.

### Additional Measures

The game software automatically generates a user log file that records all in-app activity. Each user interaction is time-stamped, allowing for calculation of time spent on specific components of the game, as well as total exposure time.

Intervention arm participants (n=27) and their parents (n=22) took part in FGDs (n=8) between T2 and T3. The four adolescent focus groups were stratified by age (11-12 and 13-14 years) and gender of the study child; the four parent focus groups were stratified by the age of the study child. Questions in postintervention discussions with participants included what they had learned from the game. Parental focus group questions also included how their children had played the game and communicated about it and with whom.

### Data Analysis

Preliminary cleaning of survey data was conducted in MS Excel, with additional cleaning and all analyses completed using SAS version 9.4 (SAS Institute Inc., Cary, NC, USA). All control arm participants were included in analyses. One participant from the intervention arm was removed from analyses of effect at T2 due to delayed completion of the T2 survey. His data were retained for T1-T3 analyses, as he completed all other study activities on time. Descriptive statistics on demographic questions and game feedback questions were computed.

Changes in behavioral mediators of sexual behavior from baseline (T1) were compared between the two study arms at T2 and T3 in an intent-to-treat analysis, using two-tailed two-sample *t* tests on individual survey items, as well as domain-level composite scores. This approach was used to identify both which theoretical mediators and which thematic areas were influenced by the intervention. Composite scores (eg, knowledge) were calculated as the equally weighted sum of the individual items within that domain (or thematic area) for which there were objectively correct or incorrect answers. In composite scores, each correct answer was worth 1 point. Analyses were conducted across the whole sample, as well as stratified by age and gender of the participants.

Data from the phone log files were downloaded as .txt files and converted into Excel files, and exposure time was calculated from time stamps. Focus group transcripts were translated into English and uploaded to MAXQDA 2018 (VERBI Software, Berlin, Germany), where they were labeled with inductive and deductive codes by two coders. The data were analyzed thematically and compared across demographics.

## Results

### Description of Study Sample

We recruited and enrolled 60 adolescent participants. Half of the participants were allocated to the intervention arm. All adolescents who were recruited completed all 3 study visits, and all intervention arm participants initiated gameplay. Participant demographics are presented in Table 3. There were no significant demographic differences between the two arms. Preliminary calculations of exposure indicate that the intervention arm played *Tumaini* a mean of approximately 27 hours over the 16 days of the intervention.

**Table 3:** Participant demographics.

Characteristics		Intervention (n=30)	Control (n=30)	Total (N=60)
**Gender, n (%)**				
	Female	14 (47)	16 (53)	30 (50)
	Male	16 (53)	14 (47)	30 (50)
Age (years), mean (SD)		12.8 (1)	12.6 (1)	12.7 (1)
**Religion, n (%)**				
	Catholic	14 (47)	14 (47)	28 (47)
	Protestant/Anglican	8 (27)	2 (7)	10 (17)
	Muslim	2 (7)	4 (13)	6 (10)
	Seventh Day Adventist	4 (13)	4 (13)	8 (13)
	Other	2 (7)	6 (20)	8 (13)
Living with both parents, n (%)		22 (73)	20 (67)	42 (70)
**Housing type, n (%)**				
	Permanent	8 (27)	13 (43)	21 (35)
	Semi-permanent	11 (37)	6 (20)	17 (28)
	Temporary	9 (30)	6 (20)	15 (25)
	Iron sheets	2 (7)	4 (13)	6 (10)
**Smartphone ownership, n (%); check all that apply**				
	Parent	21 (70)	15 (50)	36 (60)
	Self	2 (7)	1 (3)	3 (5)
	Sibling	11 (37)	5 (17)	16 (27)
	Other adult	4 (13)	1 (3)	5 (8)
	No one	3 (10)	8 (27)	11 (18)
Have used a smartphone before baseline, n (%)		22 (73)	19 (63)	41 (68)

### Behavioral Survey Outcomes

Analyses of changes in survey scores between T1 and T2 and between T1 and T3 showed a significant effect of the intervention on individual survey items and on composite scores for certain theoretical domains. Results from the two-sample *t* tests comparing changes in scores from T1 to T3 across the two study arms are reported in the following format: mean score change and SD for each study arm, t-statistic with associated degrees of freedom, and *P* value.

There was no significant difference in the overall baseline scores between the two arms: intervention arm, mean 30.73 (SD 5.32) and control arm, mean 31.13 (SD 4.74); for baseline difference, *t*_58_=0.30, *P*=.76 (see Table 4). The intervention arm saw significantly greater gains in the overall survey scores (mean 8.03, SD 4.46) than the control arm (mean 2.23, SD 3.88) at T3 (*t*_58_=−5.38, *P*<.001). At T3, the intervention arm showed significant gains in knowledge (mean 3.80, SD 2.37) compared with the control arm (mean 0.80, SD 2.14) (*t*_58_=−5.14, *P*<.001).

At T3, the intervention arm participants also showed significant sustained increases in self-efficacy scores (mean 2.03, SD 1.83) compared with the control arm (mean 0.63, SD 1.20) (t_58_=−3.50, *P*=<.001).

At baseline, participants reported having 7-8 trusted individuals they could turn to for advice. By T3, players had identified a mean of 3.10 additional sources of advice compared with 1.53 for the control arm (*t*_58_=−1.19, *P*=.24).

At T3, the intervention arm participants’ score gains for behavioral intentions for risk avoidance and reduction showed significant increases compared with those of the control arm (*t*_58_=−2.87, *P*=.006), although they had not been significant at T2. No significant change was seen in the intervention arm participants’ assessment of risk, attitudinal measures, or perceived social norms compared with the control arm.

The intervention arm showed significant increases in survey scores across constructs (eg, knowledge, attitudes, risk assessment, self-efficacy, and behavioral intentions) in the thematic areas of puberty (*t*_58_=−3.46, *P*=.001), HIV (*t*_58_=−3.25, *P*=.002), condoms (*t*_58_=−4.06, *P*=.001), and pressure from adults and peers (*t*_58_=−2.41, *P*=0.02) compared with the control arm (Table 5).

**Table 4:** Baseline scores and changes in knowledge, attitudes, intentions, risk assessment, self-efficacy, perception of social norms, sources of advice, and overall score on the behavioral survey between baseline (T1) and postintervention (T2) and between baseline and 6 weeks postintervention (T3) by study condition.

Behavioral mediator (mean change from baseline)			Number of items, maximum possible score	Intervention (n=30)		Control (n=30)	*P* value
**Knowledge, mean (SD)**			15				
	Baseline score^a^			7.33 (2.12)	7.93 (1.74)		
	T1-T2^b^			4.76^c^ (2.96)	0.27 (2.07)		<.001
	T1-T3			3.80 (2.37)	0.80 (2.14)		<.001
**Self-efficacy, mean (SD)**			9				
	Baseline score^a^			5.87 (2.03)	6.22 (2.41)		
	T1-T2^b^			1.95 (1.57)	0.47 (1.07)		<.001
	T1-T3			2.03 (1.83)	0.63 (1.20)		<.001
**Sources of advice, mean (SD)**			33				
	Baseline score^a^			7.57 (5.79)	7.07 (6.59)		
	T1-T2^b^			2.24 (4.09)	1.13 (4.19)		.31
	T1-T3			3.00 (4.79)	1.53 (4.73)		.24
**Risk assessment, mean (SD)**			4				
	Baseline score^a^			2.67 (1.25)	2.45 (1.50)		
	T1-T2^b^			0.41 (1.17)	0.00 (1.23)		.19
	T1-T3			0.52 (0.99)	0.07 (1.03)		.09
**Behavioral intention, mean (SD)**			6				
	Baseline score^a^			4.43 (0.77)	4.83 (0.70)		
	T1-T2^b^			0.28 (0.86)	−0.12 (0.76)		.07
	T1-T3			0.43 (0.75)	−0.15 (0.82)		.006
**Attitudes, mean (SD)**			15				
	Baseline score^a^			9.95 (2.30)	9.22 (2.07)		
	T1-T2^b^			0.74 (2.13)	0.82 (1.95)		.89
	T1-T3			1.18 (1.82)	0.80 (2.25)		.47
**Perceived social norms, mean (SD)**			6				
	Baseline score^a^			3.72 (1.12)	3.67 (1.24)		
	T1-T2^b^			0.47 (1.26)	−0.07 (1.30)		.12
	T1-T3			0.37 (0.37)	−0.15 (1.35)		.14
**Total score, mean (SD)**			49^d^				
	Baseline score^a^			30.73 (5.32)	31.13 (4.74)		
	T1-T2^b^			8.09 (5.78)	1.58 (3.45)		<.001
	T1-T3			8.03 (4.46)	2.23 (3.88)		<.001

^a^Mean domain score at baseline for participants in each study arm.

^b^T1-T2 calculations based on n=59.

^c^Positive values indicate a desirable change in scores.

^d^Total survey score does not include questions where there is no objectively correct or incorrect answer (sources of advice and perceived social norms).

**Table 5:** Changes in thematic domain scores (combined knowledge, attitudes, intentions, risk perception, and self-efficacy scores) on the behavioral survey between baseline (T1) and postintervention (T2) and between baseline and 6 weeks postintervention (T3) by study condition.

Thematic domains (mean change from baseline)		Number of items, maximum possible score	Intervention (n=30)	Control (n=30)	*P* value
**Future, mean (SD)**		1			
	Baseline score^a^		0.86 (0.30)	0.90 (0.24)	
	T1-T2^b^		0.05^c^ (0.34)	0.10 (0.34)	.55
	T1-T3		0.04 (0.31)	0.08 (0.27)	.54
**Puberty, mean (SD)**		4			
	Baseline score^a^		2.27 (0.98)	2.84 (0.79)	
	T1-T2^b^		0.83 (0.99)	0.18 (0.17)	.01
	T1-T3		0.86 (1.10)	−0.03 (0.94)	.002
**Alcohol or drugs, mean (SD)**		1			
	Baseline score^a^		0.77 (0.43)	0.87 (0.35)	
	T1-T2^b^		0.21 (0.41)	−0.03 (0.21)	.03
	T1-T3		0.20 (0.39)	0.03 (0.41)	.11
**Peer pressure, mean (SD)**		10			
	Baseline score^a^		7.03 (1.40)	7.02 (1.53)	
	T1-T2^b^		0.69 (1.60)	0.15 (1.35)	.17
	T1-T3		0.90 (1.26)	0.07 (1.39)	.02
**Pregnancy, mean (SD)**		2			
	Baseline score^a^		1.07 (0.78)	1.17 (0.75)	
	T1-T2^b^		0.72 (0.80)	0.03 (0.72)	<.001
	T1-T3		0.59 (1.02)	0.17 (0.87)	.09
**HIV, mean (SD)**		8			
	Baseline score^a^		4.02 (1.42)	3.83 (0.86)	
	T1-T2^b^		1.66 (1.74)	0.22 (1.45)	.001
	T1-T3		1.24 (1.44)	0.15 (1.23)	.003
**Condoms, mean (SD)**		11			
	Baseline score^a^		5.70 (2.05)	6.02 (1.83)	
	T1-T2^b^		3.17 (2.49)	0.20 (1.47)	<.001
	T1-T3		2.88 (2.20)	0.80 (1.67)	<.001
**Sex, mean (SD)**		10			
	Baseline score^a^		7.32 (1.84)	6.87 (1.45)	
	T1-T2^b^		0.66 (1.64)	0.62 (1.44)	.92
	T1-T3		1.05 (1.57)	0.95 (1.75)	.82
**Gender, mean (SD)**		3			
	Baseline score^a^		1.73 (0.73)	1.62 (0.77)	
	T1-T2^b^		0.09 (0.86)	0.12 (0.81)	.89
	T1-T3		0.14 (0.85)	0.00 (0.68)	.50

^a^Mean domain score at baseline for participants in each study arm.

^b^T1-T2 calculations based on n=59.

^c^Positive values indicate a desirable change in scores.

Analyses stratified by gender and age (11-12 year olds vs 13-14 year olds) showed similar patterns in score increases. In particular, knowledge, self-efficacy, and the thematic domain of condoms showed significant gains in all four subgroups of participants.

### Quantitative Game Experience Data

The postintervention survey eliciting participant feedback on the game revealed high subjective measures of the value, relevance, and appeal of the game, as well as participants’ perceived gains in self-efficacy to address risk situations. All participants (n=30) indicated that they had learned “a lot” and that the information would be “very useful for the future” (see Table 6). Of these participants, 29 found the information presented to be immediately useful. The overwhelming majority further responded that, after playing, they felt more prepared to handle difficult situations (n=28) and to say no firmly in situations of pressure (n=29). Ratings of the game’s appeal were very positive, with most players rating it as “very fun” (n=27) and indicating that they would like to play “much more” (n=28) and would tell their friends to play (n=29).

### Qualitative Game Experience Data

Participants’ comments and those of their parents during postintervention FGDs provided context for the gains observed in behavioral survey scores. Participants identified a wide range of knowledge and skills they had gained through playing *Tumaini*. Puberty, the reproductive systems, HIV, STIs, and condom use were mentioned repeatedly. Skills commonly mentioned were saying a strong no, how to use condoms, recognizing and avoiding bad influences, and setting and achieving goals. One female player reported, “It taught me how I can abstain from sex and how I can say a firm no to those who are persuading me to have unprotected sex and how I can keep myself away from them” (FGD for females, aged 13-14 years).

Participants reported sharing—or intending to share—what they had learned with their peers. A younger female participant said the game was useful: “If we are under pressure or forced to have sex with someone, I found that very educative and I even teach others” (FGD for females, aged 11-12 years). An older male participant felt confident he could now teach others about condom use: “*Tumaini* also teaches how to use a condom well and if [my friends] do not know how to use it I would go with them and teach them how to use a condom” (FGD for males, aged 13-14 years).

Many participants described attitudinal learning related to gender, consent, delaying sex, condom use, puberty, and people living with HIV. When asked what he thought of *Tumaini*, one older male participant responded saying, “the game taught me I do not have to force girls to do something if they do not want to” (FGD for males, aged 13-14 years). Another male participant described *Tumaini* as “the game that shows girls are as important as the boys are” (FGD for males, aged 13-14 years).

A common theme among both parent and child focus groups was the value of the game in helping children set goals and plan how to achieve them, including when faced with challenges. Parents reported that their children’s newly identified or reinforced goals were encouraging them to study hard and make good choices in order to be successful. In one child’s words, “It helps you plan your future and not make bad choices so that when you grow up you may have a smooth future and a happy family” (FGD for females, aged 11-12 years). This future orientation was presented by parents and children as a key motivator for risk avoidance or risk reduction.

Parents also described how the game had facilitated discussion about HIV and related subjects with their children. Parents reported that participants had sought out adults—parents, older siblings, and teachers—to discuss or validate the information presented in the game. One parent recalled his daughter asking, “Father, so it is true that when out there if a boy calls you to go to where he is you can refuse?” (FGD2 for parents of 13-14 year olds). Another reported, “You know at this stage men may also be interested in this young girl, and if such a thing happens right now I know she would tell me” (FGD1 for parents of 13-14 year olds).

**Table 6:** Game experience survey responses.

Variables			Male (n=16)		Female (n=14)		All (N=30)
**Value and Relevance, n (%)**							
	Learned a lot	16 (100)		14 (100)		30 (100)	
	Information very useful now	15 (94)		14 (100)		29 (97)	
	Information very useful for future	16 (100)		14 (100)		30 (100)	
	*Since playing Tumaini, I feel more prepared for difficult situations I might face in the future*	16 (100)		12 (86)		28 (93)	
	*Since playing Tumaini, I feel more sure I can say no firmly when people are trying to pressure me*	16 (100)		13 (93)		29 (97)	
**Appeal, n (%)**							
	In general playing was very fun	13 (81)		14 (100)		27 (90)	
	Would like to play much more	15 (94)		13 (93)		28 (93)	
	Would tell friends to play	15 (94)		14 (100)		29 (97)	

## Discussion

### Principal Findings

In this pilot study, we found evidence of significant effects of exposure to a game-based intervention on mediators of sexual risk avoidance and risk reduction, including related knowledge, self-efficacy, and behavioral intentions, in addition to overall survey scores at 6 weeks postintervention. This is notable and encouraging, given that this pilot study was intended only to establish the directionality of effects on behavior change and was not powered to detect changes in any behavioral mediators. Additionally, the duration of the intervention (16 days) was very brief, which may have limited its potential effects on mediators of sexual risk. Should the game prove efficacious and be available for download to parents’, older siblings’, or adolescents’ own phones, no external time limit would be placed on gameplay, thereby allowing adolescents to make use of the intervention at will, potentially maximizing its effects. Once the game is downloaded, full functionality of the game would be available without data or internet access.

FGDs with youth and parents contextualized these quantitative findings within participants’ reports of gains in knowledge and skills, increased reflection on and planning for their future, and increased dialogue with parents. The increase in the number of trusted adults identified by participants as sources of information in the surveys was also validated by parents’ focus group comments.

In the behavioral surveys, no significant effect was seen on risk assessment, attitudes, or perceived social norms. However, participants in FGDs mentioned attitudinal learning around themes including gender, consent, delaying sex, and puberty. In a systematic review and meta-analysis of sexual health interventions involving serious digital games, DeSmet et al found that changes in attitudes have not been observed [54]. However, Fiellin et al reported attitudinal changes in boys and younger participants in their recent RCT of a tablet-based HIV prevention game among US minority youth of a similar age to our study participants [55]. Narrative, which forms the central component of *Tumaini*, is considered a particularly promising way to influence attitudes [54]. While it has been argued that the effects of computer-based interventions may be stronger when nonmixed gender groups are targeted [56], *Tumaini* requires players to play characters of both sexes (and one HIV-positive character) with the aim of using empathetic identification through role-play to challenge harmful norms. A larger study, powered to detect these effects, is needed in order to better understand whether our narrative-based approach influences attitudes and norms.

High levels of intrinsic motivation among adolescents and of acceptability to parents are critical for the feasibility of a remotely delivered intervention for this age group. Several sources of evidence triangulate to support *Tumaini* ’s high appeal to participants. An objective indicator of participants’ liking of the game is mean exposure, which was over 50% higher than instructed. Enthusiasm for the game in subjective feedback provided immediately postintervention was also reflected in FGDs with participants and with parents.

### Comparison with Other Studies

HIV prevention interventions that seek to reach children before they engage in sexual risk show particular promise in improving sexual health [4,5]. In Sub-Saharan Africa, an HIV prevention intervention for 11-14 year olds found significant reductions in self-reported sexual risk behaviors compared with a control intervention 54 months postintervention [57-59]. This theoretically and contextually grounded group-based intervention, conducted in schools in South Africa, had some similarities to our intervention. It used cartoon workbooks to incorporate narrative and prepare for role-play, and activities including games, along with take-home assignments to increase parent-child communication. While *Tumaini* does not incorporate group-based activities, it is clear from the levels of discussion with parents, siblings, and peers reported in focus groups that it provoked considerable family and interpersonal interaction. In addition, as a smartphone-based intervention, *Tumaini* has certain advantages over a group-based intervention, namely the potential for sustained and on-demand exposure, increased fidelity to intervention design (because not reliant on a cadre of facilitators), low cost of implementation per participant, scalability, ease of cultural adaptability, and remotely delivered updates.

In their meta-analysis, DeSmet et al [54] found that sexual health games had positive effects, albeit small in size. However, they noted that most games in their study did not use immersive game features, relying instead on gamification features such as reward and feedback. They identify features believed to facilitate behavior change, namely tailoring, personalization, personal goal setting, narrative, scaffolding levels, challenges of increasing difficulty, interactivity, rewards, feedback, and real-life transfer. *Tumaini* incorporates all of these components, with tailoring determined by the player through the decisions made in the choose-your-own-adventure game. *Tumaini* places particularly strong emphasis on role-playing and simulation, which are believed to be especially well-suited to influencing behavioral determinants like knowledge, attitudes, skills, and self-efficacy. In the context of a larger, longer study, a mediation analysis, drawing on the game log files, will allow us to better identify the active ingredients of this game design. Results from analyses of thematic domains suggest that the game mechanics and platform are versatile and can lead to gains across a range of thematic priorities, in addition to gains across theoretical mediators.

The limitations of this study include the small sample size and limited exposure and follow-up time. A future efficacy study should track behaviors in addition to behavioral mediators and ideally include biomarkers for sexual activity to validate self-report data.

### Conclusion

To the best of our knowledge, this is the first randomized controlled study to demonstrate the influence on behavioral mediators of a smartphone game for HIV prevention in Sub-Saharan Africa. We are aware of only one other RCT of a serious sexual health game in Sub-Saharan Africa that is currently underway: this is of a mobile game designed to increase HIV risk perception among an adult population in Swaziland [60].

Our findings support the need for a rigorous study of the efficacy of *Tumaini* and similar game-based mobile interventions, with long-term follow-up and measures of behavior and not merely their determinants, ideally validated by biomarkers. Such a study could incorporate mediation analyses to pinpoint active ingredients. If appropriately grounded in behavioral theory, evidence-based practice, and contextually relevant scenarios, electronic games delivered via smartphones have the potential to become valuable tools in HIV prevention efforts in low-resource and high-prevalence settings.

## Appendix

Multimedia Appendix 1 Tumaini homescreen.

Multimedia Appendix 2 Sample graphic from narrative.

Multimedia Appendix 3 Level 1 opening screen.

Multimedia Appendix 4 CONSORT-EHEALTH checklist (V 1.6.1.).

## Abbreviations

ACASI: audio computer-assisted self-interview
CDC: US Centers for Disease Control and Prevention
FGD: focus group discussion
GEI: Gender Equity Index
GEM Scale: Gender Equitable Men Scale
KEMRI: Kenya Medical Research Institute
RCT: randomized controlled trial
STI: sexually transmitted infection
