# Supplementary figures and images for: A Smartphone Game-Based Intervention (Tumaini) to Prevent HIV Among Young Africans: Pilot Randomized Controlled Trial
Source: JMIR Mhealth Uhealth. 2018 Aug 1;6(8):e10482. doi: 10.2196/10482 (PMC6094086; doi:10.2196/10482)

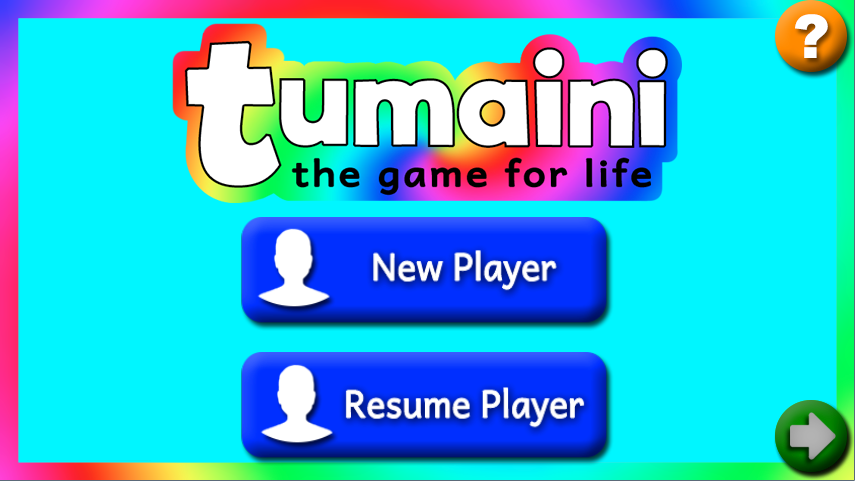

Supplement: Multimedia Appendix 1 [file mhealth_v6i8e10482_app1.png]

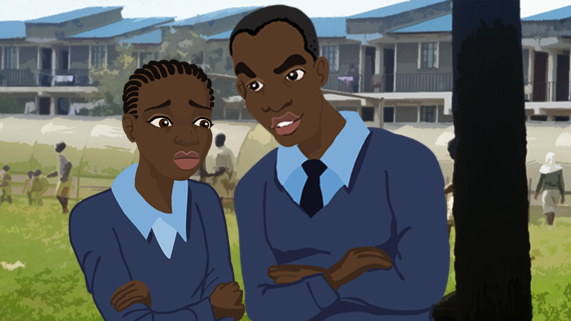

Supplement: Multimedia Appendix 2 [file mhealth_v6i8e10482_app2.png]

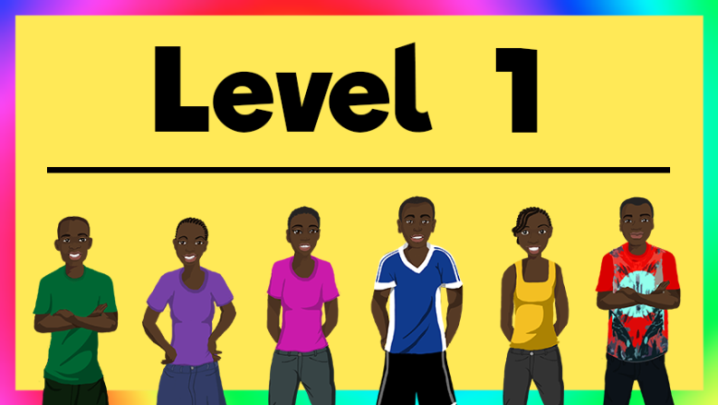

Supplement: Multimedia Appendix 3 [file mhealth_v6i8e10482_app3.png]
