# Supplementary material for: A Smartphone Game-Based Intervention (Tumaini) to Prevent HIV Among Young Africans: Pilot Randomized Controlled Trial
Source: JMIR Mhealth Uhealth. 2018 Aug 1;6(8):e10482. doi: 10.2196/10482 (PMC6094086; doi:10.2196/10482)
Supplement: Multimedia Appendix 4 [file mhealth_v6i8e10482_app4.pdf]

CONSORT-EHEALTH Checklist V1.6.2 Report	Manuscript Number	10482
(based on CONSORT-EHEALTH V1.6), available at [http://tinyurl.com/consort-ehealth-v1-6].		
**Date completed**		
7/31/2018 11:49:30		
**by**		
Kate Winskell		
A Smartphone Game-Based Intervention to Prevent HIV Among Young Africans (Tumaini): Pilot Randomized Controlled Trial		
TITLE		
**1a-i) Identify the mode of delivery in the title**		
"A Smartphone Game-Based Intervention"		
**1a-ii) Non-web-based components or important co-interventions in title**		
There were no other co-interventions or non-smartphone support		
**1a-iii) Primary condition or target group in the title**		
"to Prevent HIV Among Young Africans"		
ABSTRACT		
**1b-i) Key features/functionalities/components of the intervention and comparator in the METHODS section of the ABSTRACT**		
Tumaini ("hope for the future" in Swahili) is an interactive, narrative-based game grounded in social cognitive theory. Intervention arm participants (n=30) were provided with an Android smartphone with Tumaini installed on it and were instructed to play the game for at least 1 hour a day for 16 days; control arm participants (n=30) received no intervention.		
**1b-ii) Level of human involvement in the METHODS section of the ABSTRACT**		
"Intervention arm participants (n=30) were provided with an Android smartphone with Tumaini installed on it and were instructed to play the game for at least 1 hour a day for 16 days" - As implied here, there was no human involvement related to the intervention once participants received the devices. Participants could reach out to study staff if questions arose.		
**1b-iii) Open vs. closed, web-based (self-assessment) vs. face-to-face assessments in the METHODS section of the ABSTRACT**		
"All participants completed a survey on behavioral mediators, delivered via an audio computer-assisted self-interview system at baseline (T1), post intervention (T2), and at 6 weeks postintervention (T3)." "Intervention arm participants and their parents participated in 8 postintervention focus group discussions." Participants were recruited through school-initiated contact via letters given to eligible children inviting parents to attend informational sessions.		
**1b-iv) RESULTS section in abstract must contain use data**		
Number of participants is included in the methods: "with 60 participants<br>aged 11-14 (mean 12.7) years. Intervention arm participants (n=30)... control arm participants (n=30)"<br>"Intervention arm participants played Tumaini for a mean of approximately 27 hours."		
**1b-v) CONCLUSIONS/DISCUSSION in abstract for negative trials**		
The trial had significant findings.		
INTRODUCTION		
**2a-i) Problem and the type of system/solution**		
"A third of all new adult HIV infections occur in young people<br>aged 15-24 years [1]. In African countries most affected by<br>HIV, demographic change is increasing the size of adolescent<br>cohorts, thereby increasing their contribution to HIV incidence<br>[2]. In addition, this age group suffers disproportionately high<br>levels of HIV-related morbidity and mortality [3]. Reaching<br>preadolescents with prerisk prevention interventions may help<br>establish lifelong patterns of safer sexual behavior and avert<br>high-risk behaviors in the future" "In addition to their appeal, games for sexual health have further distinctive advantages over common group-based evidence-based interventions [29]. They have considerable potential for scalability, low cost per person reached, and cultural adaptability. Exposure to the intervention can be reliably measured through automated data-collection, which can also help pinpoint "active ingredients," contributing to the building of behavioral, pedagogical, and game design theory. Fidelity to intervention design is much more likely as the intervention is no longer dependent on a skilled cadre of facilitators. Electronic delivery offers potential for remote updates, while portability via mobile handsets can allow the intervention to link into people's everyday lives, offering more sustained intervention exposure." "There is a pressing need to assess the feasibility of using game technologies for HIV prevention in low-resource settings and their potential for efficacy". The manuscript describes a feasibility study in which a smartphone-based game was used as a stand-alone intervention. The piloted game models dialogue with trusted adults and access to services.		
**2a-ii) Scientific background, rationale: What is known about the (type of) system**		
"Electronic games have the potential to be a valuable tool in<br>youth HIV prevention in Sub-Saharan Africa if they are<br>appropriately grounded in behavioral and instructional theory<br>[8,9], informed by existing evidence-based interventions [10],<br>and contextually appropriate. Smartphone ownership is<br>increasing dramatically in emerging and developing nations<br>[11], opening up new possibilities for delivering highly<br>interactive, culturally relevant mHealth interventions at scale<br>and low cost. Serious digital games [12] have high entertainment<br>and motivational appeal for young people. They also have<br>distinctive advantages from the perspective of pedagogy and<br>behavioral theory. By allowing players to experience real agency<br>in a virtual and safe environment, well-designed games provide<br>a level of experiential learning unparalleled by many other<br>interventions. They are particularly well aligned with key<br>constructs of social cognitive theory [13], allowing for both<br>cognitive and behavioral rehearsal through role-play and<br>simulation. Although a relatively limited number of games to<br>date have been designed with solid theoretical grounding and<br>rigorously evaluated [14-20], there is evidence of their<br>effectiveness for health, including clinical outcomes [21-28]." The choice of comparator (no intervention) is justified in the Methods section: it reflects current standard of care.		
**Does your paper address CONSORT subitem 2b?**		
"There is a pressing need to assess the feasibility of using game<br>technologies for HIV prevention in low-resource settings and<br>their potential for efficacy. In this study, we pilot-tested an<br>interactive narrative-based smartphone game to prevent HIV<br>among preadolescents in Kisumu Town, Western Kenya, where<br>adult HIV prevalence (19.9%) is over three times the national<br>average [30,31]. We describe here results from this pilot study<br>of the game's potential to influence behavioral mediators of<br>increased age and condom use at sexual debut."		
METHODS		
**3a) CONSORT: Description of trial design (such as parallel, factorial) including allocation ratio**		
"We conducted an individually randomized pilot study of the<br>game Tumaini" "Participants (n=60) were randomized 1:1 to the control arm<br>(n=30) or the intervention (game) arm (n=30) of the study."		
**3b) CONSORT: Important changes to methods after trial commencement (such as eligibility criteria), with reasons**		
There were no changes after trial commencement.		
**3b-i) Bug fixes, Downtimes, Content Changes**		
There were no changes after study commencement.		
**4a) CONSORT: Eligibility criteria for participants**		
"The eligibility criteria for participation were as follows: age<br>11-14 years, grade 3-4 English proficiency on the<br>Flesch-Kincaid Reading Scale, residence in Kisumu Town, and<br>willingness to complete all study activities."		
**4a-i) Computer / Internet literacy**		

This was a feasibility study.		
**4a-ii) Open vs. closed, web-based vs. face-to-face assessments:**		
"Letters were distributed through schools to parents of age-eligible children<br>inviting them to attend informational meetings."<br>"All participants completed a self-administered behavioral survey<br>at T1, T2, and T3. The English language survey was completed<br>at the KEMRI offices, using the audio computer-assisted<br>self-interview (ACASI) system with headphones to protect<br>privacy. It took approximately 1 hour to complete."<br>"Intervention arm participants (n=27) and their parents (n=22) took part in FGDs (n=8) between T2 and T3."<br>"Data from the phone log files were downloaded as .txt files and<br>converted into Excel files,"		
**4a-iii) Information giving during recruitment**		
"Letters were distributed through schools to parents of age-eligible children<br>inviting them to attend informational meetings. Consent and<br>assent were secured at the home of the participants, following<br>an explanation of the study. Parents consented to participate in<br>the postintervention focus groups if their child was randomized<br>to the intervention arm." Informational meetings included general information about the potential of the game and the problems it aims to address as well as a description of the study. Consent procedures reviewed this content.<br>"Assignments were revealed to participants after they had<br>completed the baseline assessment"<br>"Intervention arm participants completed a 45-minute<br>informational onboarding session, including instructions on the<br>interface, technology, and game content."<br>Parents of intervention arm participants also attended an onboarding session and received the same information as their children about the game's content.		
**4b) CONSORT: Settings and locations where the data were collected**		
"The English language survey was completed<br>at the KEMRI offices, using the audio computer-assisted<br>self-interview (ACASI) system with headphones to protect<br>privacy." The manuscript indicates that participants also took part in FGDs- these were carried out at the KEMRI offices and in the offices of local CBOs.		
**4b-i) Report if outcomes were (self-)assessed through online questionnaires**		
The survey was carried out via ACASI in person. Data was also downloaded from the study phones. There was no online questionnaire component.		
**4b-ii) Report how institutional affiliations are displayed**		
The participants were told about institutional affiliations during recruitment and consenting, and study activities occurred at the KEMRI offices.		
**5) CONSORT: Describe the interventions for each group with sufficient details to allow replication, including how and when they were actually administered**		
**5-i) Mention names, credential, affiliations of the developers, sponsors, and owners**		
"game for inexpensive Android smartphones developed in collaboration with a US commercial game developer, Realtime Associates, and with input from US-based and Kenyan specialists in adolescent sexual health and Kenyan preadolescents and their parents."<br>"Research reported in this publication was supported by the National Institute of Mental Health of the US National Institutes of Health under Award Number 5R34MH106368 (PI: KW)."		
**5-ii) Describe the history/development process**		
This is a feasibility study so it is part of the development process. Additional information included in this paper: "developed in collaboration with a US commercial game developer, Realtime Associates, and with input from US-based<br>and Kenyan specialists in adolescent sexual health and Kenyan<br>preadolescents and their parents."		
**5-iii) Revisions and updating**		
There were no revisions or updates during the trial		
**5-iv) Quality assurance methods**		
The game was developed "with input from US-based and Kenyan specialists in adolescent sexual health"		
**5-v) Ensure replicability by publishing the source code, and/or providing screenshots/screen-capture video, and/or providing flowcharts of the algorithms used**		
We provide illustrative screenshots.		
**5-vi) Digital preservation**		
The app will eventually be made available if proven efficacious. At this stage, it is not online nor widely accessible. Only study participants are provided with the intervention.		
**5-vii) Access**		
Intervention participants were provided with a phone with<br>the game preloaded and used it at their own pace for the duration<br>of the intervention.		
**5-viii) Mode of delivery, features/functionalities/components of the intervention and comparator, and the theoretical framework**		
The game is "a theoretically grounded,<br>narrative-based game for inexpensive Android smartphones<br>developed in collaboration with a US commercial game<br>developer, Realtime Associates, and with input from US-based<br>and Kenyan specialists in adolescent sexual health and Kenyan<br>preadolescents and their parents."<br>"The game's design draws on social behavioral theory, including<br>social cognitive theory [13] and the theory of possible selves<br>[32]; existing evidence-based interventions for youth HIV<br>prevention [33,34-36]; and games for health [8,12,17,37] and<br>entertainment-education [38] literature. It is grounded in research<br>on HIV-themed narratives written by young Africans [39-41].<br>Tumaini uses interactive narrative to promote observational<br>learning, cognitive and behavioral rehearsal, problem-solving,<br>and immersion."<br>The game is made up of 3 intersecting components: (1) a role-playing narrative; (2) a set of mini-games; (3) a self-reflection component. The topics of (2) and (3) are tied to the narrative.		
**5-ix) Describe use parameters**		
"They were instructed to play at least 1 hour per day for the 16 days of the study and asked not to share their own gameplay profile with others."<br>Participants "used it at their own pace for the duration of the intervention."		
**5-x) Clarify the level of human involvement**		
Participants and their parents were told to reach out to study staff with any questions that arose as needed.		
**5-xi) Report any prompts/reminders used**		
An alarm set to ring once a day reminded players to play.		
**5-xii) Describe any co-interventions (incl. training/support)**		
There were no co-interventions. "Intervention arm participants completed a 45-minute informational onboarding session, including instructions on the interface, technology, and game content".		
**6a) CONSORT: Completely defined pre-specified primary and secondary outcome measures, including how and when they were assessed**		

This paper presents data on secondary outcomes only. Primary outcomes are discussed in a separate publication.<br><br>"All participants completed a self-administered behavioral survey at T1, T2, and T3. The English language survey was completed at the KEMRI offices, using the audio computer-assisted self-interview (ACASI) system."<br><br>"The behavioral survey assessed mediators associated with age at onset of sexual activity and condom use at sexual debut, including knowledge, self-efficacy, risk assessment, perceived social norms, attitudes, and behavioral intentions. Thematic areas included puberty, sex, relationships, peer pressure, condom use, HIV, STIs, pregnancy, and alcohol and drugs."<br><br>"The game software automatically generates a user log file that records all in-app activity. Each user interaction is time-stamped, allowing for calculation of time spent on specific components of the game, as well as total exposure time."<br><br>"Intervention arm participants (n=27) and their parents (n=22) took part in FGDs (n=8) between T2 and T3. The four adolescent focus groups were stratified by age (11-12 and 13-14 years) and gender of the study child; the four parent focus groups were stratified by the age of the study child. Questions in postintervention discussions with participants included what they had learned from the game. Parental focus group questions also included how their children had played the game and communicated about it and with whom."<br><br>"Preliminary cleaning of survey data was conducted in MS Excel, with additional cleaning and all analyses completed using SAS version 9.4 (SAS Institute Inc., Cary, NC, USA). All control arm participants were included in analyses. One participant from the intervention arm was removed from analyses of effect at T2 due to delayed completion of the T2 survey. His data were retained for T1-T3 analyses, as he completed all other study activities on time. Descriptive statistics on demographic questions and game feedback questions were computed. Changes in behavioral mediators of sexual behavior from baseline (T1) were compared between the two study arms at T2 and T3 in an intent-to-treat analysis, using two-tailed two-sample t tests on individual survey items, as well as domain-level composite scores. This approach was used to identify both which theoretical mediators and which thematic areas were influenced by the intervention. Composite scores (eg, knowledge) were calculated as the equally weighted sum of the individual items within that domain (or thematic area) for which there were objectively correct or incorrect answers. In composite scores, each correct answer was worth 1 point. Analyses were conducted across the whole sample, as well as stratified by age and gender the participants."<br>"Data from the phone log files were downloaded as .txt files and converted into Excel files, and exposure time was calculated from time stamps. Focus group transcripts were translated into English and uploaded to MAXQDA 2018 (VERBI Software, Berlin, Germany), where they were labeled with inductive and deductive codes by two coders. The data were analyzed thematically and compared across demographics."		
**6a-i) Online questionnaires: describe if they were validated for online use and apply CHERRIES items to describe how the questionnaires were designed/deployed**		
Not applicable		
**6a-ii) Describe whether and how "use" (including intensity of use/dosage) was defined/measured/monitored**		
"The game software automatically generates a user log file that records all in-app activity. Each user interaction is time-stamped, allowing for calculation of time spent on specific components of the game, as well as total exposure time."<br><br>"Data from the phone log files were downloaded as .txt files and converted into Excel files, and exposure time was calculated from time stamps."		
**6a-iii) Describe whether, how, and when qualitative feedback from participants was obtained**		
"Intervention arm participants (n=27) and their parents (n=22) took part in FGDs (n=8) between T2 and T3. The four adolescent focus groups were stratified by age (11-12 and 13-14 years) and gender of the study child; the four parent focus groups were stratified by the age of the study child. Questions in postintervention discussions with participants included what they had learned from the game. Parental focus group questions also included how their children had played the game and communicated about it and with whom."<br>"Focus group transcripts were translated into English and uploaded to MAXQDA 2018 (VERBI Software, Berlin, Germany), where they were labeled with inductive and deductive codes by two coders. The data were analyzed thematically and compared across demographics."		
**6b) CONSORT: Any changes to trial outcomes after the trial commenced, with reasons**		
"The English language survey was completed at the KEMRI offices, using the audio computer-assisted self-interview (ACASI) system with headphones to protect privacy." The manuscript indicates that participants also took part in FGDs- these were carried out at the KEMRI offices and in the offices of local CBOs.		
**7a) CONSORT: How sample size was determined**		
**7a-i) Describe whether and how expected attrition was taken into account when calculating the sample size**		
This is not included in the manuscript as it is a feasibility trial and it was not powered to detect effects of the intervention, but merely intended to assess directionality of effect.		
**7b) CONSORT: When applicable, explanation of any interim analyses and stopping guidelines**		

This paper presents data on secondary outcomes only. Primary outcomes are discussed in a separate publication.<br><br>"All participants completed a self-administered behavioral survey at T1, T2, and T3. The English language survey was completed at the KEMRI offices, using the audio computer-assisted self-interview (ACASI) system."<br><br>"The behavioral survey assessed mediators associated with age at onset of sexual activity and condom use at sexual debut, including knowledge, self-efficacy, risk assessment, perceived social norms, attitudes, and behavioral intentions. Thematic areas included puberty, sex, relationships, peer pressure, condom use, HIV, STIs, pregnancy, and alcohol and drugs."<br><br>"The game software automatically generates a user log file that records all in-app activity. Each user interaction is time-stamped, allowing for calculation of time spent on specific components of the game, as well as total exposure time."<br><br>"Intervention arm participants (n=27) and their parents (n=22) took part in FGDs (n=8) between T2 and T3. The four adolescent focus groups were stratified by age (11-12 and 13-14 years) and gender of the study child; the four parent focus groups were stratified by the age of the study child. Questions in postintervention discussions with participants included what they had learned from the game. Parental focus group questions also included how their children had played the game and communicated about it and with whom."<br><br>"Preliminary cleaning of survey data was conducted in MS Excel, with additional cleaning and all analyses completed using SAS version 9.4 (SAS Institute Inc., Cary, NC, USA). All control arm participants were included in analyses. One participant from the intervention arm was removed from analyses of effect at T2 due to delayed completion of the T2 survey. His data were retained for T1-T3 analyses, as he completed all other study activities on time. Descriptive statistics on demographic questions and game feedback questions were computed. Changes in behavioral mediators of sexual behavior from baseline (T1) were compared between the two study arms at T2 and T3 in an intent-to-treat analysis, using two-tailed two-sample t tests on individual survey items, as well as domain-level composite scores. This approach was used to identify both which theoretical mediators and which thematic areas were influenced by the intervention. Composite scores (eg, knowledge) were calculated as the equally weighted sum of the individual items within that domain (or thematic area) for which there were objectively correct or incorrect answers. In composite scores, each correct answer was worth 1 point. Analyses were conducted across the whole sample, as well as stratified by age and gender the participants."<br>"Data from the phone log files were downloaded as .txt files and converted into Excel files, and exposure time was calculated from time stamps. Focus group transcripts were translated into English and uploaded to MAXQDA 2018 (VERBI Software, Berlin, Germany), where they were labeled with inductive and deductive codes by two coders. The data were analyzed thematically and compared across demographics."		
**8a) CONSORT: Method used to generate the random allocation sequence**		
"Randomization, stratified by the school attended by the participant, gender, and age, was undertaken using a coin flip by a blinded research team member. Within each school, gender, and age block of participants, coin flips were repeated until participants were equally distributed between the two study arms"		
**8b) CONSORT: Type of randomisation; details of any restriction (such as blocking and block size)**		
"Randomization, stratified by the school attended by the participant, gender, and age, was undertaken using a coin flip by a blinded research team member. Within each school, gender, and age block of participants, coin flips were repeated until participants were equally distributed between the two study arms"		
**9) CONSORT: Mechanism used to implement the random allocation sequence (such as sequentially numbered containers), describing any steps taken to conceal the sequence until interventions were assigned**		
"Assignments were revealed to participants after they had completed the baseline assessment."		
**10) CONSORT: Who generated the random allocation sequence, who enrolled participants, and who assigned participants to interventions**		
"Randomization [...] was undertaken [...] by a blinded research team member"<br>Enrollment procedures were undertaken by other team members. Allocation was revealed by team members not involved in randomization but involved in enrollment.		
**11a) CONSORT: Blinding - If done, who was blinded after assignment to interventions (for example, participants, care providers, those assessing outcomes) and how**		
**11a-i) Specify who was blinded, and who wasn't**		
This was not a blinded study		
**11a-ii) Discuss e.g., whether participants knew which intervention was the "intervention of interest" and which one was the "comparator"**		
The control group received no intervention so all involved knew which was "the intervention of interest".		
**11b) CONSORT: If relevant, description of the similarity of interventions**		
Not applicable		
**12a) CONSORT: Statistical methods used to compare groups for primary and secondary outcomes**		
"Descriptive statistics on demographic questions and game feedback questions were computed. Changes in behavioral mediators of sexual behavior from baseline (T1) were compared between the two study arms at T2 and T3 in an intent-to-treat analysis, using two-tailed two-sample t tests on individual survey items, as well as domain-level composite scores."		
**12a-i) Imputation techniques to deal with attrition / missing values**		
There was no attrition.		
**12b) CONSORT: Methods for additional analyses, such as subgroup analyses and adjusted analyses**		
"Changes in behavioral mediators of sexual behavior from baseline (T1) were compared between the two study arms at T2 and T3 in an intent-to-treat analysis, using two-tailed two-sample t tests on individual survey items, as well as domain-level composite scores.[...] Analyses were conducted across the whole sample, as well as stratified by age and gender the participants."		
RESULTS		

**13a) CONSORT: For each group, the numbers of participants who were randomly assigned, received intended treatment, and were analysed for the primary outcome**		
"We recruited and enrolled 60 adolescent participants. Half of<br>the participants were allocated to the intervention arm." Also, see Figure 1.		
**13b) CONSORT: For each group, losses and exclusions after randomisation, together with reasons**		
There were none. See Figure 1.		
**13b-i) Attrition diagram**		
The study phones were collected after the end of the intervention period so this is not applicable here.		
**14a) CONSORT: Dates defining the periods of recruitment and follow-up**		
This is included in the methods: "We conducted an individually randomized pilot study of the game Tumaini ("hope for the future" in Swahili) in a sample of<br>60 male and female preadolescents aged 11-14 years in<br>periurban and urban Kisumu, Kenya, between April and June<br>2017. The intervention was carried out over 16 days during the<br>3-week school holiday in April 2017 (Figure 1). Assessment<br>was performed via a survey at baseline (T1), immediately<br>postintervention (T2), and at 6 weeks postintervention (T3).<br>Intervention arm participants also took part in focus group<br>discussions (FGDs) after the intervention to provide additional<br>data on the game experience"<br>Recruitment took place in March 2017.		
**14a-i) Indicate if critical "secular events" fell into the study period**		
N/A		
**14b) CONSORT: Why the trial ended or was stopped (early)**		
N/A		
**15) CONSORT: A table showing baseline demographic and clinical characteristics for each group**		
This is included as Table 3- Participant demographics		
**15-i) Report demographics associated with digital divide issues**		
Participants were asked about smartphone ownership and use. However, access was not an issue for this study since smartphones were provided.		
**16a) CONSORT: For each group, number of participants (denominator) included in each analysis and whether the analysis was by original assigned groups**		
**16-i) Report multiple "denominators" and provide definitions**		
Analyses were based on intent-to-treat assignments. All participants were included in baseline and endline analyses. One participant in the intervention group was excluded from postintervention analyses. This is explained in Tables 4 and 5. All intervention participants initiated gameplay, therefore ITT also represented ToT analyses.		
**16-ii) Primary analysis should be intent-to-treat**		
Yes. In methods: "Changes in behavioral mediators of sexual behavior from<br>baseline (T1) were compared between the two study arms at T2<br>and T3 in an intent-to-treat analysis,"		
**17a) CONSORT: For each primary and secondary outcome, results for each group, and the estimated effect size and its precision (such as 95% confidence interval)**		
Only secondary outcomes are included here as primary outcomes were not related to behavioral outcomes and are presented elsewhere. Results are presented in tables 4 and 5.<br>"There was no significant difference in the overall baseline scores<br>between the two arms: intervention arm, mean 30.73 (SD 5.32)<br>and control arm, mean 31.13 (SD 4.74); for baseline difference,<br>t58=0.30, P=.76 (see Table 4). The intervention arm saw<br>significantly greater gains in the overall survey scores (mean<br>8.03, SD 4.46) than the control arm (mean 2.23, SD 3.88) at T3<br>(t58=−5.38, P<.001). At T3, the intervention arm showed<br>significant gains in knowledge (mean 3.80, SD 2.37) compared<br>with the control arm (mean 0.80, SD 2.14) (t58=−5.14, P<.001).<br>At T3, the intervention arm participants also showed significant<br>sustained increases in self-efficacy scores (mean 2.03, SD 1.83)<br>compared with the control arm (mean 0.63, SD 1.20) (t58=−3.50,<br>P=<.001).<br>At baseline, participants reported having 7-8 trusted individuals<br>they could turn to for advice. By T3, players had identified a<br>mean of 3.10 additional sources of advice compared with 1.53<br>for the control arm (t58=−1.19, P=.24).<br>At T3, the intervention arm participants' score gains for<br>behavioral intentions for risk avoidance and reduction showed<br>significant increases compared with those of the control arm<br>(t58=−2.87, P=.006), although they had not been significant at<br>T2. No significant change was seen in the intervention arm<br>participants' assessment of risk, attitudinal measures, or<br>perceived social norms compared with the control arm.<br>The intervention arm showed significant increases in survey<br>scores across constructs (eg, knowledge, attitudes, risk<br>assessment, self-efficacy, and behavioral intentions) in the<br>thematic areas of puberty (t58=−3.46, P=.001), HIV (t58=−3.25,<br>P=.002), condoms (t58=−4.06, P=.001), and pressure from adults<br>and peers (t58=−2.41, P=0.02) compared with the control arm<br>(Table 5).<br>Analyses stratified by gender and age (11-12 year olds vs 13-14<br>year olds) showed similar patterns in score increases. In<br>particular, knowledge, self-efficacy, and the thematic domain<br>of condoms showed significant gains in all four subgroups of<br>participants.<br>Quantitative Game Experience Data<br>The postintervention survey eliciting participant feedback on<br>the game revealed high subjective measures of the value,<br>relevance, and appeal of the game, as well as participants'<br>perceived gains in self-efficacy to address risk situations. All<br>participants (n=30) indicated that they had learned "a lot" and<br>that the information would be "very useful for the future" (see<br>Table 6). Of these participants, 29 found the information<br>presented to be immediately useful. The overwhelming majority<br>further responded that, after playing, they felt more prepared to<br>handle difficult situations (n=28) and to say no firmly in<br>situations of pressure (n=29). Ratings of the game's appeal were<br>very positive, with most players rating it as "very fun" (n=27)<br>and indicating that they would like to play "much more" (n=28)<br>and would tell their friends to play (n=29)."		
**17a-i) Presentation of process outcomes such as metrics of use and intensity of use**		
"Preliminary calculations of exposure indicate that the<br>intervention arm played Tumaini a mean of approximately 27<br>hours over the 16 days of the intervention."		
**17b) CONSORT: For binary outcomes, presentation of both absolute and relative effect sizes is recommended**		
N/A		
**18) CONSORT: Results of any other analyses performed, including subgroup analyses and adjusted analyses, distinguishing pre-specified from exploratory**		
The paper indicates that stratified analyses showed similar patterns, however these are not presented due to the small strata sizes		
**18-i) Subgroup analysis of comparing only users**		
All intended users initiated gameplay.		
**19) CONSORT: All important harms or unintended effects in each group**		

None were detected or reported.		
**19-i) Include privacy breaches, technical problems**		
N/A		
**19-ii) Include qualitative feedback from participants or observations from staff/researchers**		
"Qualitative Game Experience Data<br>Participants' comments and those of their parents during postintervention FGDs provided context for the gains observed in behavioral survey scores. Participants identified a wide range of knowledge and skills they had gained through playing Tumaini. Puberty, reproductive systems, HIV, STIs, and condom use were mentioned repeatedly. Skills commonly mentioned were saying a strong no, how to use condoms, recognizing and avoiding bad influences, and setting and achieving goals. One female player reported "It taught me how I can abstain from sex and how I can say a firm no to those who are persuading me to have unprotected sex and how I can keep myself away from them" (FGD for females, aged 13-14 years).<br>Participants reported sharing—or intending to share—what they had learned with their peers. A younger female participant said the game was useful: "If we are under pressure or forced to have sex with someone, I found that very educative and I even teach others" (FGD for females, aged 11-12 years). An older male participant felt confident he could now teach others about condom use: "Tumaini also teaches how to use a condom well and if [my friends] do not know how to use it I would go with them and teach them how to use a condom" (FGD for males, aged 13-14 years).<br>Many participants described attitudinal learning related to gender, consent, delaying sex, condom use, puberty, and people living with HIV. When asked what he thought of Tumaini, one older male participant responded saying, "the game taught me I do not have to force girls to do something if they do not want to" (FGD for males, aged 13-14 years). Another male participant described Tumaini as "the game that shows girls are as important as the boys are" (FGD for males, aged 13-14 years).<br>A common theme among both parent and child focus groups was the value of the game in helping children set goals and plan how to achieve them, including when faced with challenges. Parents reported that their children's newly identified or reinforced goals were encouraging them to study hard and make good choices in order to be successful. In one child's words, "It helps you plan your future and not make bad choices so that when you grow up you may have a smooth future and a happy family" (FGD for females, aged 11-12 years). This future orientation was presented by parents and children as a key motivator for risk avoidance or risk reduction.<br>Parents also described how the game had facilitated discussion about HIV and related subjects with their children. Parents reported that participants had sought out adults—parents, older siblings, and teachers—to discuss or validate the information presented in the game. One parent recalled his daughter asking, "Father, so it is true that when out there if a boy calls you to go to where he is you can refuse?" (FGD2 for parents of 13-14 year olds). Another reported, "You know at this stage men may also be interested in this young girl, and if such a thing happens right now I know she would tell me" (FGD1 for parents of 13-14 year olds)."		
DISCUSSION		
**20) CONSORT: Trial limitations, addressing sources of potential bias, imprecision, multiplicity of analyses**		
**20-i) Typical limitations in ehealth trials**		
"The limitations of this study include the small sample size and limited exposure and follow-up time. A future efficacy study should track behaviors in addition to behavioral mediators and ideally include biomarkers for sexual activity to validate self-report data."		
**21) CONSORT: Generalisability (external validity, applicability) of the trial findings**		
**21-i) Generalizability to other populations**		
This is a feasibility study.		
**21-ii) Discuss if there were elements in the RCT that would be different in a routine application setting**		
"the duration of the intervention (16 days) was very brief, which may have limited its potential effects on mediators of sexual risk. Should the game prove efficacious and be available for download to parents', older siblings', or adolescents' own phones, no external time limit would be placed on gameplay, thereby allowing adolescents to make use of the intervention at will, potentially maximizing its effects. Once the game is downloaded, full functionality of the game would be available without data or internet access."		
**22) CONSORT: Interpretation consistent with results, balancing benefits and harms, and considering other relevant evidence**		
**22-i) Restate study questions and summarize the answers suggested by the data, starting with primary outcomes and process outcomes (use)**		

"In this pilot study, we found evidence of significant effects of exposure to a game-based intervention on mediators of sexual risk avoidance and risk reduction, including related knowledge, self-efficacy, and behavioral intentions, in addition to overall survey scores at 6 weeks postintervention."<br><br>"FGDs with youth and parents contextualized these quantitative findings within participants' reports of gains in knowledge and skills, increased reflection on and planning for their future, and increased dialogue with parents. The increase in the number of trusted adults identified by participants as sources of information in the surveys was also validated by parents' focus group comments."<br><br>"In the behavioral surveys, no significant effect was seen on risk assessment, attitudes, or perceived social norms. However, participants in FGDs mentioned attitudinal learning around themes including gender, consent, delaying sex, and puberty."<br><br>"High levels of intrinsic motivation among adolescents and of acceptability to parents are critical for the feasibility of a remotely delivered intervention for this age group. Several sources of evidence triangulate to support Tumaini 's high appeal to participants. An objective indicator of participants' liking of the game is mean exposure, which was over 50% higher than instructed. Enthusiasm for the game in subjective feedback provided immediately postintervention was also reflected in FGDs with participants and with parents."		
**22-ii) Highlight unanswered new questions, suggest future research**		
This study was a feasibility study and is intended to inform a larger scale RCT to show efficacy of the intervention.<br><br>We note, among other things: "A larger study, powered to detect these [changes in attitudes and social norms] effects, is needed in order to better understand whether our narrative-based approach influences attitudes and norms" and "In the context of a larger, longer study, a mediation analysis, drawing on the game log files, will allow us to better identify the active ingredients of this game design."<br>"A future efficacy study should track behaviors in addition to behavioral mediators and ideally include biomarkers for sexual activity to validate self-report data."		
Other information		
**23) CONSORT: Registration number and name of trial registry**		
The study "was registered with ClinicalTrials.gov (NCT03054051)."		
**24) CONSORT: Where the full trial protocol can be accessed, if available**		
This will be available as a future publication.		
**25) CONSORT: Sources of funding and other support (such as supply of drugs), role of funders**		
"Acknowledgments<br>Research reported in this publication was supported by the National Institute of Mental Health of the US National Institutes of Health under Award Number 5R34MH106368 (PI: KW). The content is solely the responsibility of the authors and does not necessarily represent the official views of the National Institutes of Health. This research was also supported by the Emory Center for AIDS Research (P30 AI050409) and the Andrew W. Mellon Foundation. The sponsors paid no role in review and approval of the manuscript for publication."		
**X26-i) Comment on ethics committee approval**		
"The study was approved by the Emory University and Kenya Medical Research Institute (KEMRI) Institutional Review Boards"		
**x26-ii) Outline informed consent procedures**		
"Consent and assent were secured at the home of the participants, following an explanation of the study. Parents consented to participate in the postintervention focus groups if their child was randomized to the intervention arm." Additional details will be provided in a separate protocol manuscript: consent was required from one parent at least and the child's assent was also secured for successful enrollment. Staff reviewed the project, responded to questions and rescreened for eligibility prior to consent and assent procedures.		
**X26-iii) Safety and security procedures**		
In developing the survey: "A draft instrument was presented to parents for acceptability, then cognitively tested in 3 rounds with preadolescents to ensure acceptability, consistent interpretation, and face validity of the questions."<br>The survey was delivered "using the audio computer-assisted self-interview (ACASI) system with headphones to protect privacy."<br>Gameplay log files were only identified by the participant's study ID, as were survey responses. FGDs were also coded without identifying participant information.<br>Participants and parents were told to contact study staff with any concerns. Study staff were trained to recognize signs of distress during survey taking and to provide counseling and linkage to services if needed.		
**X27-i) State the relation of the study team towards the system being evaluated**		
The study sponsor is NIMH. DW led the development of the app for Realtime Associates. The game is owned by Emory University.		
